# Causal relationship between gastroesophageal reflux disease and 7 types of hernias: A bidirectional Mendelian randomization study

**DOI:** 10.1097/MD.0000000000044251

**Published:** 2025-09-05

**Authors:** Xunsheng Chen, Jingyi Chen, Qingling Yin, Meijun Hou, Xueqing Xie, Wei Lu, Jingjing Tian, Ke Zhang, Jie Ding

**Affiliations:** a School of Clinical Medicine, Guizhou Medical University, Guiyang, Guizhou Province, China; b Department of Gastrointestinal Surgery, Guizhou Provincial People’s Hospital, Guiyang, Guizhou Province, China; c Shengli Clinical Medical College, Fujian Medical University, Fuzhou, Fujian Province, China; d Guizhou University Medical College, Guiyang, Guizhou Province, China; e School of Clinical Medicine, Zunyi Medical University, Zunyi, Guizhou Province, China; f Department of Hepatobiliary Surgery, Yunmeng People’s Hospital, Xiaogan, Hubei Province, China.

**Keywords:** gastroesophageal reflux disease, genetic epidemiology, genome-wide association study, hernia, Mendelian randomization

## Abstract

Gastroesophageal reflux disease (GERD) is linked to various esophageal and extra-esophageal complications. While GERD is theoretically a potential risk factor for abdominal hernias, current evidence is limited. Observational studies have suggested associations between GERD and both congenital diaphragmatic hernia and hiatal hernia. This study employed a bidirectional Mendelian randomization analysis to assess the causal relationship between GERD and 7 types of abdominal hernias. The inverse variance weighting method served as the primary statistical approach, supplemented by sensitivity analyses. Inverse variance weighting results demonstrated significant causal relationships between GERD and increased risks of 5 types of hernias: abdominal wall hernia (odds ratio [OR]: 1.277, 95% confidence interval [CI]: 1.144–1.425, *P* < .001), umbilical hernia (OR: 1.270, 95% CI: 1.086–1.485, *P* < .01), incisional hernia (OR: 1.484, 95% CI: 1.261–1.748, *P* < .001), diaphragmatic hernia (OR: 1.333, 95% CI: 1.171–1.518, *P* < .001), and hiatal hernia (OR: 1.015, 95% CI: 1.012–1.018, *P* < .001). No causal links were found with inguinal or femoral hernia. Reverse Mendelian randomization analysis revealed that both diaphragmatic hernia (OR: 1.119, 95% CI: 1.077–1.162, *P* < .001) and hiatal hernia (OR: 609.061, 95% CI: 83.659–4434.156, *P* < .001) were causally associated with an increased risk of GERD. Other hernia types showed no significant reverse association. These findings support a bidirectional causal relationship between GERD and specific hernias and may inform improved strategies for diagnosis, treatment, and prevention.

## 1. Introduction

Gastroesophageal reflux disease (GERD) is a prevalent global condition characterized by the reflux of gastric contents into the esophagus, resulting in symptoms such as acid reflux and chest pain.^[1]^ With a global prevalence of approximately 13%, GERD exhibits geographic variability.^[2]^ Several risk factors have been associated with GERD, including obesity, smoking, genetic predisposition, and physical labor.^[3,4]^ The symptoms of GERD can be categorized into esophageal and non-esophageal. Oesophageal complications include stenosis of the digestive tract, Barrett’s esophagus and esophageal adenocarcinoma. Non-esophagus effects can impact various organs, including the heart, oropharynx, and lungs.^[1]^ GERD is associated with an increased risk of lung diseases, such as chronic cough, chronic obstructive pulmonary disease (COPD), and asthma, and may also increase intra-abdominal pressure.^[5]^ The high prevalence and multisystem impact of GERD have led to diverse therapeutic strategies, including lifestyle modifications, pharmacological treatments, endoscopic therapies, and surgical interventions, all aimed at relieving symptoms and minimizing long-term complications.^[6]^

A hernia refers to the protrusion of an organ through a weakness or defect in the tendon membrane that encases the organ.^[7]^ Abdominal wall hernias occur when an organ protrudes through a weakened area in the abdominal wall and include inguinal hernia, umbilical, femoral, and incisional hernias.^[8]^ Diaphragmatic hernias occur when abdominal organs enter the thoracic cavity through a defect in the diaphragm and can be classified as congenital or traumatic.^[9]^ Observational studies indicate that survivors of congenital diaphragmatic hernia (CDH) have an elevated risk of GERD, though the underlying mechanisms remain unclear. Potential contributing factors include esophageal dyskinesia, esophageal shortening, diaphragmatic weakness, disruption of the angle of His, intestinal torsion, local anatomical alterations and increased intra-abdominal pressure postsurgical repair.^[10–12]^ Hiatal hernia, a subtype of diaphragmatic hernia, involves the protrusion of abdominal contents, typically the stomach, through the diaphragm into the mediastinum. Symptoms often include gastroesophageal reflux.^[13,14]^ the pathogenesis of hernias is complex and involves congenital factors, aging, obesity, smoking and increased intra-abdominal pressure from activities such as heavy lifting or coughing.^[7,15,16]^ Although GERD is hypothesized to induce increased abdominal pressure, thus increasing the risk of hernias, there is a lack of studies investigating the effect of GERD on abdominal hernias. Research into the causal relationship between GERD and hernias is essential for improving diagnosis and prevention strategies.

Mendelian randomization (MR) is a methodological approach used in epidemiological research to infer causal relationships between phenotypes using genetic variation as an instrumental variable. A commonly used form of genetic variation is the single nucleotide polymorphism (SNP), which refers to the substitution of a single base pair at a specific position in the genome that commonly occurs within the population.^[17]^ In MR analysis, SNPs that are strongly associated with a trait of interest can serve as instrumental variables for subsequent causal inference studies. Based on Mendel’s second law, which states that individual genotypes are randomly inherited, MR can mitigate confounding factors and reverse causality in observational studies.^[18]^ Genome-wide association studies (GWAS) provides a rich source of SNPs associated with complex traits and diseases, offering valid instruments for MR analysis.^[19]^ Therefore, this study employs MR to investigate the potential causal relationship between GERD and 7 types of hernias, aiming to advance research in these areas and offer a theoretical foundation for clinical disease management.

## 2. Materials and methods

### 2.1. Study design

**Figure 1.**
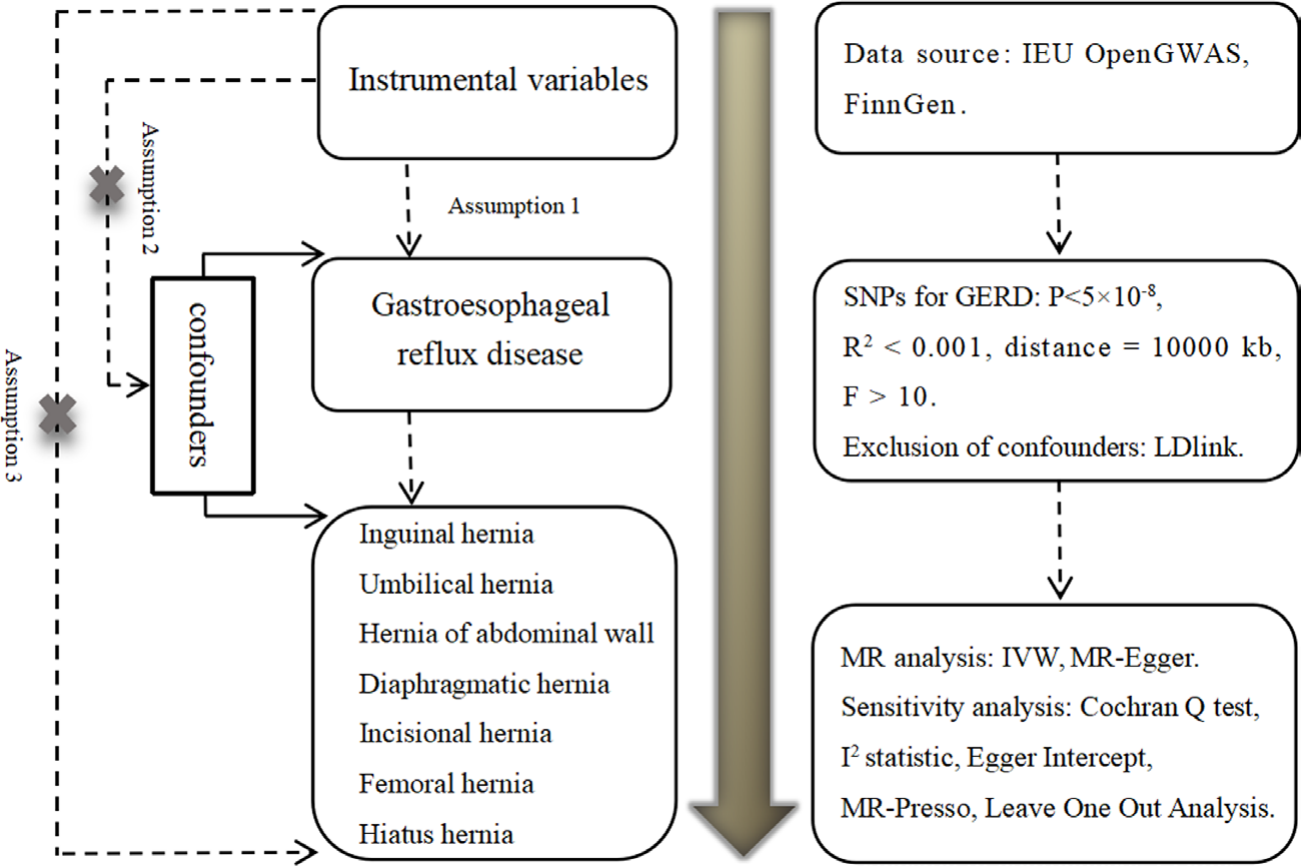
Flow chart of forward Mendelian randomization analysis. Assumption 1: Relevance assumption, Assumption 2: Independence assumption, Assumption 3: Exclusion restriction assumption. GERD = gastroesophageal reflux disease, GWAS = genome-wide association study, IEU = Integrative Epidemiology Unit, IVW = inverse variance weighting, LD = linkage disequilibrium, MR-PRESSO = Mendelian randomization Pleiotropy Residual Sum and Outlier, SNPs = single nucleotide polymorphisms.

To investigate the causal relationship between GERD and 7 types of hernias, we conducted a 2-sample MR study. First, we performed a forward MR analysis using GERD as the exposure and the 7 hernia types as outcomes to evaluate the causal effect of GERD on the development of these hernias. Then, we conducted a reverse MR analysis using the hernias as exposures and GERD as the outcome to explore the potential causal effects of these hernias on GERD. Figure 1 illustrates the basic principles of MR and the study flow for forward MR analysis.

### 2.2. Data source

GERD data were obtained from a publicly available GWAS in the Integrative Epidemiology Unit (IEU) OpenGWAS database (https://gwas.mrcieu.ac.uk/), under the ID: ebi-a-GCST90000514. The GWAS data included 1,29,080 patients with GERD and 4,73,524 individuals from European populations, with data provided by Ong JS.^[20]^ The hiatal hernia GWAS data were also sourced from IEU OpenGWAS (ID: ukb-b-6514), encompassing 10,662 patients with hiatal hernia and 4,52,271 Europeans, with data provided by the MRC-IEU Committee and Ben Elsworth.

The FinnGen study, a large-scale genomics initiative initiated in 2017, analyzed over 5,00,000 Finnish biobank samples to correlate genetic variations with health data. Detailed information is available on the FinnGen website (https://www.finngen.fi/fi). The GWAS data for abdominal wall hernia, inguinal hernia, umbilical hernia, femoral hernia, incisional hernia, and diaphragmatic hernia were obtained from FinnGen, as outlined in Table 1. Statistical analyses were conducted using the TwoSampleMR and Mendelian Randomization Pleiotropy Residual Sum and Outlier (MR-PRESSO) R packages in R version 4.3.2.

**Table 1:** Details of the GWAS database for gastroesophageal reflux disease and 7 types of hernias.

Phenotype	Consortium	Author	ID	Cases/controls	Ancestry
GERD	NA	Ong JS	ebi-a-GCST90000514	1,29,080/4,73,524	European
Inguinal hernia	FinnGen	NA	K11_HERING	35,248/3,52,418	European
Umbilical hernia	FinnGen	NA	K11_UMBHER	9403/3,52,418	European
Hernia of abdominal wall	FinnGen	NA	ABDOM_HERNIA	16,839/3,95,342	European
Diaphragmatic hernia	FinnGen	NA	K11_DIAHER	12,886/3,52,418	European
Incisional hernia	FinnGen	NA	ABDOM_HERNIA_POSTOP	6336/23,2612	European
Femoral hernia	FinnGen	NA	K11_FEMHER	1349/3,52,418	European
Hiatus hernia	MRC-IEU	Ben Elsworth	ukb-b-6514	10,662/4,52,271	European

GERD = gastroesophageal reflux disease, GWAS = genome-wide association study.

### 2.3. Instrumental variables selection

The selection of SNPs as instrumental variables was guided by 3 basic assumptions of MR: the assumption of relevance: the selected genetic variants must be correlated with the exposure; the assumption of independence: the selected genetic variants should not be associated with any confounding factors; and the assumption of exclusion: the selected genetic variants must not be directly associated with the outcome.^[21]^

To select SNPs as instrumental variables for GERD, we applied the following stringent criteria to ensure validity and robustness of the MR analysis: Relevance: SNPs were required to be strongly associated with GERD at genome-wide significance (*P*-value < 5 × 10^−8^). Linkage disequilibrium (LD) clumping: To minimize effects, we applied a clumping threshold of *R*^2^ < 0.001 with a physical distance of 10,000 kilobases (kb). Exclusion of confounders: SNPs associated with potential confounding factors such as obesity and smoking were removed using LDlink (https://ldlink.nih.gov/?tab=ldtrait).^[22]^ Strand ambiguity avoidance: Palindromic SNPs were excluded to avoid strand alignment errors and ensure result robustness. Instrument strength: The *F*-statistic for each SNP was calculated using the formula *F* = *R*^2^ × (N − 2) ÷ (1 − *R*^2^). Only SNPs with *F* > 10 were retained to avoid weak instrument bias.^[23]^ Reverse MR – For the reverse MR analysis, SNP selection followed similar criteria as in the forward analysis.

### 2.4. MR analysis

This study utilized a 2-sample MR to explore the causal relationship between GERD and 7 types of hernias. The primary analytical method used in this study was inverse variance weighting (IVW). This method first calculates the Wald ratio for each SNP and then combines them using a weighted average, where each SNP is weighted by the inverse of its variance. As a result, SNPs with smaller variances have greater influence on the overall estimate, thereby improving the accuracy and efficiency of causal inference.^[24]^ For this study, a random effects model was used, as it accounts for potential heterogeneity and provides a more accurate estimation of the overall causal effect.^[24]^ The results are presented as ORs with 95% CIs. In addition, MR-Egger regression was also employed in this study. This method considers the possibility that some genetic variants may influence the outcome through pathways other than the exposure, and introduces an intercept term into the regression model to detect such bias; if the intercept is significantly different from zero, it indicates the presence of directional pleiotropy, while the slope can reflect the causal effect of the exposure on the outcome.^[25]^ To control for multiple comparisons and reduce the risk of false positives, the Bonferroni-Holm correction method was applied. Additionally, to examine the effect of the 7 types of hernias on GERD, reverse MR analyses were conducted with the hernias as exposures and GERD as the outcome.

### 2.5. Sensitivity analysis

In the selection of instrumental variables, we excluded obesity- and smoking-related SNPs to minimize the impact of potential confounders on the outcome. However, the possibility of unidentified confounding factors, which can lead to deviations from the calculation results through pleiotropy, remains. Several sensitivity analyses were performed to assess the robustness of the outcome. First, heterogeneity among SNPs was evaluated using Cochran’s *Q* test, and the strength of heterogeneity was quantified by calculating *I*-squared statistic (*I*^2^).^[26]^ Second, the presence of potential pleiotropy was assessed by examining the MR-Egger intercept term.^[25]^ Outliers were identified and removed using MR-PRESSO, and subsequent analyses were conducted without these outliers. Additionally, a leave-one-out analysis was performed to assess the influence of individual SNPs on the overall outcome. The results of the MR analysis were also visualized using a forest plot.

### 2.6. Ethics statement

The GWAS data used in this Mendelian randomized study were publicly available, and all original studies included in this study were approved by the relevant ethical review boards and informed consent was obtained from all participants. In addition, no individual-level data were used in this study and no additional ethical review board approval was required.

## 3. Results

### 3.1. Selection of instrumental variables

In this study, we identified a total of 80 SNPs associated with GERD that met the criteria of *P* < 5 × 10^−8^ and were in accordance with the LD threshold (*R*^2^ < 0.001, physical distance = 10,000 kb). The *F* values for these SNPs were calculated, and all SNPs demonstrated sufficient strength as strong instrumental variables (*F* > 10), effectively mitigating potential outcome bias due to weak instrumental variables (Table S1, Supplemental Digital Content, https://links.lww.com/MD/P850). To ensure the assumption of independence, SNPs associated with obesity and smoking – common risk factors for both GERD and hernia – were excluded as potential confounders (Table S2, Supplemental Digital Content, https://links.lww.com/MD/P850). The selected SNPs were not directly associated with hernias, consistent with the exclusivity assumption. We cross-referenced SNPs that met the basic assumption of MR with the GWAS database for abdominal wall hernia, inguinal hernia, umbilical hernia, femoral hernia, incisional hernia and diaphragmatic hernia. SNP rs2106353 was excluded due to its absence in the database, and palindromic SNPs were removed as described in Table S3, Supplemental Digital Content, https://links.lww.com/MD/P850. Ultimately, 54 SNPs were identified for the MR analysis involving GERD and abdominal wall hernia, inguinal hernia, umbilical hernia, femoral hernia, incisional hernia and diaphragmatic hernia. For the hiatal hernia, all relevant SNPs were present in the GWAS database, leading to the identification of 55 SNPs for MR analysis of GERD and hiatal hernia.

### 3.2. MR analysis

**Figure 2.**
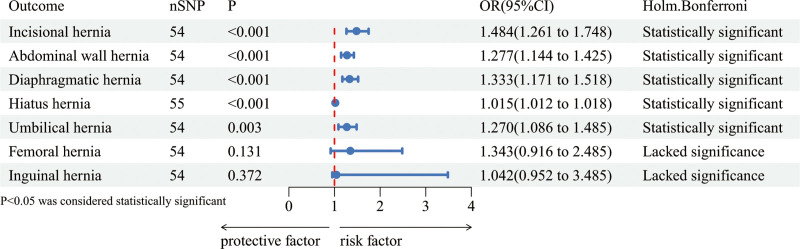
Mendelian randomization forest plot of gastroesophageal reflux disease and 7 types of hernias. CI = confidence interval, OR = odds ratio, SNP = single nucleotide polymorphism.

To investigate the direct causal relationship between GERD and the 7 types of hernias, we performed a 2-sample MR analysis using IVW as the primary method. The analysis revealed a significant association between GERD and several types of hernias: incisional hernia (odds ratio (OR): 1.484, 95% CI: 1.261–1.748, *P* < .001), abdominal wall hernia (OR: 1.277, 95% CI: 1.144–1.425, *P* < .001), diaphragmatic hernia (OR: 1.333, 95% CI: 1.171–1.518, *P* < .001), hiatal hernia (OR: 1.015, 95% CI: 1.012–1.018, *P* < .001), and umbilical hernia (OR: 1.270, 95% CI: 1.086–1.485, *P* < .001). These results indicate a significant positive correlation between GERD and these hernias. Conversely, no statistically significant correlations were observed between GERD and femoral hernia (OR: 1.343, 95% CI: 0.916–2.485, *P* = .131) or inguinal hernia (OR: 1.042, 95% CI: 0.952–3.485, *P* = .372). MR-Egger analysis further supported the significant association between GERD and hiatal hernia (OR: 1.020, 95% CI: 1.0016–1.0382, *P* < .05). However, the correlation between GERD and other hernias was not statistically significant. This may be due to the MR-Egger method’s more conservative statistical approach, which accounts for potential pleiotropy, thereby possibly reducing statistical precision.^[25]^ Finally, to ensure the robustness of the results and reduce the false positive rate of multiple testing, the Holm-Bonferroni correction was applied. The corrected results aligned with the initial findings, as detailed in Figure 2.

### 3.3. Sensitivity analysis

To ensure the robustness of the MR analysis results, we conducted multiple sensitivity analyses. First, Cochran’s *Q* test was used to assess the heterogeneity of SNPs. We did not find statistically significant heterogeneity in the MR analyses of GERD with diaphragmatic hernia, hiatal hernia, abdominal wall hernia, femoral hernia and incisional hernia. However, heterogeneity was observed in the analyses of inguinal hernia and umbilical hernia, with *I*^2^ of 30% and 27%, respectively. Despite this, the random effects IVW model used in our analysis can accommodate a certain degree of heterogeneity. Next, MR-Egger regression was employed to evaluate horizontal pleiotropy. The MR-Egger intercept term did not indicate statistically significant pleiotropy, suggesting that the observed heterogeneity did not bias the MR results. Additionally, MR-PRESSO was used to identify and exclude outliers. Outliers were detected in the analysis of inguinal hernias; however, even after removing the outliers, no statistically significant association was found. A leave-one-out sensitivity analysis was performed to assess the influence of individual SNPs on the overall outcome; no single SNP was found to significantly affect the outcome (Table S4, Supplemental Digital Content, https://links.lww.com/MD/P850).

### 3.4. Reverse MR analysis

**Figure 3.**
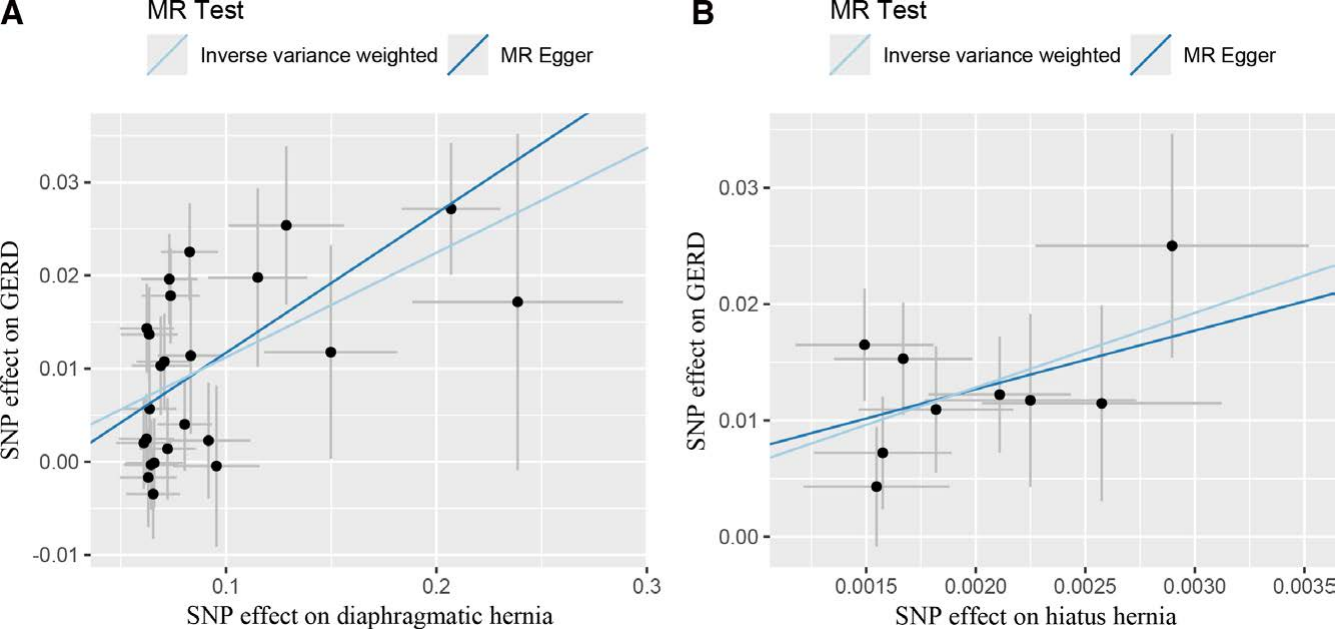
(A) Diaphragmatic hernia and GERD: A Mendelian randomization analysis scatter plot. (B) Hiatal hernia and GERD: A Mendelian randomization analysis scatter plot. GERD = gastroesophageal reflux disease, MR = Mendelian randomization, SNP = single nucleotide polymorphism.

To investigate the causal role of the 7 types of hernias on GERD, we performed a reverse MR analysis. The methodology was similar to that of the forward MR analysis. We screened SNPs that met *P* < 5 × 10^−8^, *R*^2^ < 0.001 and a physical distance of 10,000 kb to serve as instrumental variables. Initially, there were insufficient variables for incisional, femoral, diaphragmatic and hiatal hernia. Therefore, we relaxed the values as *P* < 5 × 10^−6^ and excluded SNPs not present in the GERD GWAS data, as well as SNPs related to obesity and smoking and palindromic SNPs. Ultimately, we identified 24, 9, 14, 34, 9, 5 and 6 SNPs for diaphragmatic hernia, hiatal hernia, abdominal wall hernia, inguinal hernia, umbilical hernia, femoral hernia and incisional hernia, respectively, for reverse MR analysis (Table S5, Supplemental Digital Content, https://links.lww.com/MD/P850). Notably, diaphragmatic hernia was found to increase the risk of GERD, with significant results observed in both IVW (OR: 1.119, 95% CI: 1.077–1.162, *P* < .001) and MR-Egger analyses (OR: 1.162, 95% CI: 1.039–1.298, *P* < .05). To ensure the robustness of the results, we performed multiple sensitivity analyses. Cochran’s *Q* test indicated significant heterogeneity, with an *I*^2^ value of 47%. Similarly, MR-Egger did not reveal statistically significant pleiotropy, and MR-PRESSO did not detect outliers. The leave-one-out analysis did not identify any single SNP with a significant influence on the outcomes. The study did not find a significant causal relationship between abdominal wall hernia, inguinal hernia, umbilical hernia, femoral hernia or incisional hernia and GERD. The heterogeneity observed in the MR analysis of the association between diaphragmatic hernia and GERD may be attributable to variability in the effects of different types of diaphragmatic hernias on GERD. Consequently, we performed a separate MR analysis focusing specifically on hiatal hernia and GERD. Notably, this specific type of diaphragmatic hernia significantly increased the risk of GERD, with a significant increase in IVW (OR: 609.061, 95% CI: 83.659–4434.156, *P* < .001; Fig. 3). Sensitivity analyses for this specific hernia type showed no significant heterogeneity, no pleiotropy, no outliers, and no single SNP with a significant impact on the outcome.

## 4. Discussion

To the best of our knowledge, no studies have previously examined the effect of GERD on abdominal hernias. Existing observational studies on diaphragmatic hernia and hiatal hernia and their impact on GERD may be subject to confounding biases. In this study, we performed a 2-way MR analysis using data from the FinnGenn and IEU Open GWAS databases. We found that GERD is associated with an increased risk of several hernias, including abdominal wall hernia, incisional hernia, umbilical hernia, femoral hernia, diaphragmatic hernia, and hiatal hernia. Notably, reverse Mendelian analysis indicated that diaphragmatic hernia also increases the risk of GERD. Moderate heterogeneity was also observed, which might be attributed to the varying effects of different categories of diaphragmatic hernia on GERD. Therefore, we further performed an MR analysis focusing on a subtype of diaphragmatic hernia – hiatal hernia – and found a causal relationship between hiatal hernia and increased risk of GERD. Sensitivity analyses confirmed the robustness of our results.

GERD is a common disease associated with upper gastrointestinal motility disorders. Its pathogenesis involves impaired anti-reflux mechanisms and increased reflux injury, including dysfunction of the lower esophageal sphincter, anatomical defects of the esophagus, diminished esophageal clearance, and hiatal hernia.^[27]^ Hernias of the abdominal wall encompass inguinal, umbilical, femoral and incisional hernias.^[8]^ Inguinal hernias can be lateral, protruding through the internal inguinal ring, or medial, emerging through a weak area in Hesselbach’s triangle. Risk factors for inguinal hernias include intra-abdominal high pressure, age, connective tissue alterations, constipation, genetic susceptibility and gender.^[28]^ Umbilical hernia involves protrusions through the umbilical or periumbilical opening, with congenital cases resulting from failure of umbilical ring closure and acquired cases associated with pregnancy, obesity, ascites or large intra-abdominal tumors.^[29]^ Femoral hernias occur when intra-abdominal organs pass through the femoral ring into the femoral canal below the deep iliac ligament and lateral pubic tubercle. A variety of factors, such as constipation, bronchitis and pregnancy-induced increased intra-abdominal pressure, influence the development of femoral hernias. Approximately 40% of femoral hernias are diagnosed late and require urgent surgical management, which increases mortality rates.^[30,31]^ Incisional hernias result from protrusion through surgical incision, with increased risk associated with obesity, emergency surgery, smoking, abdominal aortic aneurysm, or postoperative trauma infections.^[32]^ Diaphragmatic hernia is the entry of abdominal organs into the thoracic cavity due to a weak or defective diaphragm, encompassing congenital and traumatic types.^[9]^ Hiatal hernia, a type of diaphragmatic hernia, involves the protrusion of abdominal contents, usually the stomach, through the diaphragm into the mediastinum, including sliding hernia and transverse esophageal hernia.^[14]^

GERD extends beyond the esophagus, potentially leading to gastrointestinal complications such as Barrett’s esophagus, and esophageal adenocarcinoma, and it can also impact various organs including the heart, oropharynx and lungs.^[1]^ GERD may contribute to increased intra-abdominal pressure by increasing the risk of conditions like chronic cough, COPD, and asthma.^[5]^ When gastroesophageal reflux occurs, gastric contents may enter the respiratory tract via the esophagus and pharynx. Gastric acid and pepsin can damage the airway epithelium, induce airway hyperresponsiveness, and upregulate proinflammatory cytokines.^[33]^ In addition, acidic stimulation of the esophagus may trigger vagally mediated reflexes, leading to bronchospasm or enhanced airway responsiveness.^[5]^ These pathological changes can cause or exacerbate respiratory diseases such as chronic cough, asthma, and COPD.^[5,33]^These respiratory conditions, in turn, can elevate intra-abdominal pressure through repeated contraction of the abdominal or diaphragmatic muscles.^[34]^ Under elevated intra-abdominal pressure, abdominal organs may protrude through weak spots in the abdominal wall, leading to herniation. Protrusion upward through diaphragmatic weak points or the esophageal hiatus may result in diaphragmatic or hiatal hernias, while forward protrusion through weakened areas of the abdominal wall can lead to inguinal, femoral, umbilical, or incisional hernias. Although there is speculation that GERD may increase the risk of hernia through increased abdominal pressure due to lung diseases, there is a lack of direct studies examining this relationship. Investigating the causal link between GERD and hernia is clinically valuable for improving diagnosis, treatment and prevention strategies for both conditions.

Diaphragmatic hernia, including congenital and traumatic forms, has been associated with chronic respiratory disease, neurodevelopmental problems, neurosensory hearing loss, and gastroesophageal reflux.^[9,35]^ For instance, Caruso Anna Maria et al performed pH monitoring testing on 36 children with CDH, revealing that 83% of children with CDH exhibited gastroesophageal reflux at 6 months of age, with 62% showing GER symptoms. At 5 years, the incidence of GERD was 61%, and 38% had GER symptoms, although the incidence and symptoms decreased with age but remained above normal levels.^[36]^ Yokota Kazuki et al reported that 37.8% of CDH survivors had GERD,^[37]^ and Terui Keita et al found that 23.8% of CDH infants required pharmacologic treatment for GERD, and 10.7% underwent surgery.^[38]^ The increased GERD risk in CDH survivors is likely related to esophageal dysmotility, esophageal shortening, diaphragmatic weakness, disruption of the angle of His, intestinal torsion, as well as localized anatomical alterations and increased intra-abdominal pressure post-surgery.^[10–12]^ Hiatal hernia, a type of diaphragmatic hernia, has been strongly associated with GERD. David Ott et al conducted a retrospective study of 319 patients with upper respiratory digestive symptoms and found that hiatal hernia increased the risk of GERD.^[39]^ Nurten Savas et al studied 40 patients with hiatal hernia and observed that 27 (67.5%) of these patients had esophageal acid reflux on 24-hour pH monitoring.^[40]^ The risk of GERD with hiatal hernia may be due to mechanisms such as the separation between the lower esophageal sphincter and the esophageal hiatus, which can impair acid clearance and increase reflux incidence.^[13]^

Our study is innovative and scientifically robust. We investigated the causal relationship between GERD and various types of hernias using GWAS data from the FinnGen and IEU databases. Moreover, the GWAS data used in this study were from European populations and characterized by large sample sizes, which increased the credibility of the results. MR analysis offers advantages over traditional observational studies by reducing biases from confounding factors and reverse causality. We also conducted multiple sensitivity analyses, including MR-Egger regression, MR-PRESSO, and leave-one-out analysis, to ensure result robustness. Additionally, the bidirectional MR approach allowed for a comprehensive evaluation of interactions between GERD and several types of hernias. Nevertheless, this study has certain limitations. While it explored causal relationships, it did not investigate the underlying mechanisms. The study’s population was primarily European, which may affect the generalizability to other ethnic groups. Meanwhile, the GWAS data also did not allow for age and gender stratification. Despite excluding SNPs related to obesity and smoking, other unknown confounders may still bias the results.

In the future, further mechanistic studies are needed to elucidate the pathophysiological pathways linking GERD to the development of various types of hernias. In addition, further clinical research is required to validate these causal relationships. These efforts may ultimately contribute to more appropriate preventive strategies and therapeutic approaches for patients with gastroesophageal reflux and hernias.

## 5. Conclusion

Our bidirectional MR study explored the causal relationship between GERD and 7 types of abdominal hernias. These findings suggest that clinicians should consider the possible coexistence of abdominal wall hernias when diagnosing and managing patients with GERD, and adopt appropriate treatment strategies. Likewise, in patients with diaphragmatic hernias, especially hiatal hernias, the potential occurrence of abdominal wall hernias should be taken into account, and suitable diagnostic and therapeutic approaches should be developed accordingly.

## Acknowledgments

The authors thank Bullet Edits Limited for the linguistic editing and proofreading of the manuscript.

## Author contributions

**Conceptualization:** Xunsheng Chen, Jie Ding.

**Data curation:** Xunsheng Chen, Jingyi Chen.

**Formal analysis:** Xunsheng Chen, Jingyi Chen.

**Funding acquisition:** Jie Ding.

**Investigation:** Qingling Yin, Jingjing Tian.

**Methodology:** Xunsheng Chen, Jie Ding.

**Project administration:** Meijun Hou, Jie Ding.

**Resources:** Meijun Hou, Wei Lu.

**Visualization:** Qingling Yin, Xueqing Xie.

**Writing – original draft:** Xunsheng Chen, Jingyi Chen.

**Writing – review & editing:** Qingling Yin, Ke Zhang, Jie Ding.

## Supplementary Material


